# Identity construction among deaf adolescents and young adults: A literature review

**DOI:** 10.4102/ajod.v12i0.1168

**Published:** 2023-05-02

**Authors:** Lieketseng V. Sekoto, Vera-Genevey Hlayisi

**Affiliations:** 1 Division of Communication Sciences and Disorders, Department of Rehabilitation Sciences, Faculty of Health Sciences, University of Cape Town, Cape Town, South Africa

**Keywords:** adolescents, deaf identity, identity construction, young adults, hearing loss, disability, self-ascribed deaf disability

## Abstract

**Background:**

Identity construction is an integral developmental task for adolescents and young adults (AYA). The intersection of deaf identity and disabling hearing loss (DHL) adds a layer to the complex process of identity construction.

**Aim:**

This literature review highlights the self-ascribed deaf identities of AYA and seeks to understand how AYA with DHL forge these identities. Knowledge areas for prospective research and practice are uncovered.

**Method:**

A traditional literature review of qualitative empirical evidence on AYA’s accounts of their deaf identity construction was conducted on seminal literature and peer-reviewed journals in psychology, disability studies and deaf studies.

**Results:**

The emerging self-ascribed deaf identities of AYA are diverse. The identities include Deaf, hearing, hard-of-hearing (HOH), bicultural HOH, identities that detach from disability, bicultural DeaF, unresolved and fluid identities. Complex trade-offs exist where the construction of certain identities forgoes certain reasonable accommodations, interventions or relations that are critical for personal development and wellbeing.

**Conclusion:**

Current literature orients deaf identity formation around hearing status and Deaf-hearing communal dynamics. In-depth research comprising facets of AYA’s personal, enacted and relational identities is required to conscientise rehabilitation professionals about the nuances of deaf identity issues and how to develop interventions that are supportive and responsive to the clinical and psychosocial challenges of AYA with DHL.

**Contribution:**

This paper deviates from the d/Deaf identity dichotomy, revealing a spectrum of deaf identities that AYA forge. The rationales of AYA’s deaf identities, underlying processes and possible vulnerable identities are unpacked. Recommendations for prospective research pertaining to identity construction among deaf AYA are made.

## Introduction

### The conjunction of identity and disabling hearing loss

The World Health Organization (WHO 2021:40) categorises disabling hearing loss (DHL) as hearing loss (HL) of moderate severity or more (>35 dB) in the better hearing ear. The presence of DHL can be a drive for the personal adjustment that implicitly affects one’s evaluation of their identity. Thus, a pivotal psychosocial task that is implicated in the context of DHL is identity construction. Identity is a complex concept denoting one’s understanding of who they are. Social scientists describe identity as self-concepts resulting from the interaction of self and society (Israelite, Ower & Goldstein 2002:134). The periods of adolescence and young adulthood for individuals in the 10–25 years age range are peak developmental stages for identity construction. These periods are also characterised by rapid physical, psychological, emotional and social changes, to which adolescents and young adults (AYA) must adapt (Ozdemir, Utkualp & Palloş 2016:717).

In hearing healthcare, AYA are becoming the focal point for audiological interventions as the prevalence and risk of acquiring DHL in this population are on the rise. Globally, HL is the third leading condition that accounts for years lived with disability (Global Burden of Disease 2019 Hearing Loss Collaborators 2021:1002). Across the world, 34 million children aged 0–14 years require rehabilitation services for DHL, with most congenital and acquired paediatric DHL cases being attributed to sub-Saharan Africa (Adedeji et al. 2015:1625; Desalew et al. 2020:2). For the young people affected by DHL, societal and attitudinal barriers can be a hindrance to function across social, psychological, educational and vocational spheres, affecting participation and overall wellbeing (WHO 2021:1).

Evidently, DHL affects psychosocial wellbeing, implicating identity construction among other processes. The purpose of audiological rehabilitation is to improve communication function and psychosocial wellbeing. As modern-day audiological rehabilitation shifts from traditional medical models to more person-centred approaches, psychosocial aspects such as understanding who the person receiving care is in the context of DHL are critical. Although underexplored, research shows that identity issues relating to DHL can disrupt the provision and uptake of audiological interventions (Clark et al. 2020:55). Therefore, insight on deaf identity construction is valuable for achieving holistic care that is responsive to the unique needs of AYA.

### Understanding the construction of identity

Throughout adolescence and young adulthood, individuals actively consolidate ideations about themselves and their surroundings (Kemmery & Compton 2014:159). The resultant product is identity, a unique and distinct concept of who they are to themselves and others (Upreti 2017:54). Identity construction results from a selective acceptance and rejection of childhood identities, community identities and self-identities (Erikson 1968:159). Many theories about identity formation have been formulated. Erik Erikson’s Theory of Psychosocial Development (1968), James Marcia’s Ego Identity Status Model (1966) and Hetch’s Communication Theory of Identity (1993) are outlined in this article. These theories and model frame identity construction as a critical aspect of psychosocial development for AYA and communication as a key requisite for identity formation, thus appealing to core aspects of audiological rehabilitation.

Together, these identity formation theories capture the essence of audiological rehabilitation, which lies in improving psychosocial and communication function for persons with DHL. Thus, they lay a foundational framework through which to understand deaf identity construction among AYA with DHL. Through their background as developmental psychologists, Erikson and Marcia’s theories speak to psychosocial aspects and highlight exploration as a key process in identity construction. Marcia’s theory further edifies Erikson’s theory by providing a means to classify one’s identity status and any associated challenges. As communication scientists, Hetch and colleagues’ Communication Theory of Identity depicts the layered nature of identity and frames anew the understanding of communication function by emphasising communication as a means to identity construction.

#### Erik Erikson’s theory of psychosocial development

Erikson (1968) theorised several conflicting psychosocial states that introduce a crisis along one’s lifespan from infancy to old adulthood. The crisis of identity versus role confusion is most predominant in adolescence and young adulthood, creating a push factor for initiating identity construction (Erikson 1968:131; Upreti 2017:54). Often, identity formation has long-term implications on the social circles, health behaviours, career and vocational aspirations that AYA have (Sawyer et al. 2012:1631). Adolescents and young adults who do not resolve their identity crisis tend to plunge into instability, negative behaviours and an inability to assume and sustain adulthood roles and responsibilities (Erikson 1968). To achieve an identity, AYA need the freedom to explore different roles and identities. Further research investigated this notion of exploration and commitment in identity development.

#### James Marcia’s ego identity status model

As an expansion on Erikson’s theory, Marcia deduced four identity statuses, namely identity achievement, moratorium, foreclose and identity diffusion (Marcia 1966:557). Each identity status contrasts the relationship between exploration and commitment. Individuals attain identity achievement after extensive exploration, while those who remain in moratorium are still experimenting with roles and have not made firm commitments (Kroger et al. 2010:683). In foreclosure, individuals settle with an identity prior any kind of exploration, and lastly, those in identity diffusion are not actively exploring and do not have any commitments (Kroger et al. 2010:683). Of these statuses, moratorium is the least stable; AYA who get stuck in moratorium will present with negative behavioural patterns, psychopathology and have less intent with their social roles and vocational choices (Kroger et al. 2010:684). In diffusion, AYA isolate themselves and refrain from forming relationships (Sugimura & Mizokami 2012:126). It is evident that the process of identity formation can be turbulent, and that exploration is a fundamental requisite for identity construction.

#### Communication theory of identity

It can be argued that the ability to communicate facilitates exploration. In fact, one theory posits that through communication identity is constructed, and the communication itself is identity (Jung & Hecht 2004:266). Communication theory of identity is rooted in intercultural communication research and explores the relationship between identity and communication (Jung & Hecht). Researchers proposed four frames or layers of identity, namely personal, relational, enacted, and communal identity (Jung & Hecht).

Personal identity is constructed at an individual level as an outcome of one’s self-perception (Pang & Hutchinson 2018:21). Relational identity is drawn from a kinship with others and can be segmented into four levels. Firstly, one can construct an identity from how others view them (Jung & Hecht 2004:266). Secondly, relationships with others such as being a parent or sister, can be adopted as identities. Thirdly, relational identities can be an amalgamation of multiple interacting identities, where one is a student, prefect, athlete, daughter and youth leader at the same time (Jung & Hecht 2004:266–267). Lastly, a relation itself may constitute as an identity, as is the case where people identify as friends or a couple (Jung & Hecht 2004:266–267; Pang & Hutchinson 2018:22). Enacted identities are constructed through social behaviours and activities expressing identity, as seen in the manner which one conducts themselves (Jung & Hecht 2004:266). Communal identities emerge from affiliations with a collective or group with which one has shared values, such as racial groups, religious groups or special needs groups (Pang & Hutchinson 2018:23). In essence, through daily exchanges of information, identities are communicated with other people, and the communication shapes identities. While the abovementioned theories are insightful, they do not speak explicitly to deaf identity. Exploration of deaf identity formation literature is required to get in-depth understanding.

This literature review lies at the intersection of three concepts, identity construction, disability and HL among AYA. Little is known about how these concepts converge during adolescence and young adulthood. It seeks to highlight and understand the self-ascribed deaf identities that AYA with DHL construct. Notably, understanding identity construction is a good starting point for contextualising audiological rehabilitation interventions to the periods of adolescence and young adulthood to support identity construction as a critical psychosocial task. Therefore, this review will outline existing knowledge around deaf identity construction among AYA with DHL. Existing knowledge gaps, the implications for audiological practice and opportunities for further research will be highlighted.

## Methods

This literature review explored existing literature on deaf identity construction in the context of DHL among AYA. Database searches on PubMed, PsycINFO, Google Scholar and hand searches on seminal literature and key peer-reviewed journals in psychology, deaf studies and disability studies were perused to review qualitative research articles. Searches considered literature from 2002 to 2022 and key search terms included ‘identity construction’, ‘deaf identity’ and ‘deaf ‘adolescents’. As this was a traditional literature review that was exploratory in nature, searches were not confined to prescribed protocols. The studies selected for review were not contextualised to any geographical setting to broaden the scope of the review and were qualitative in nature consisting mainly of ethnographic and phenomenological studies as these explored elaborate accounts from AYA themselves, of their self-ascribed deaf identities.

## Review findings

### The dichotomous perception of deaf identity in the context of disabling hearing loss

Traditionally, persons with HL are thought to assume one of two identities, Deaf or deaf (McIlroy & Storbeck 2011:495). The former identity is constructed on the principles of Deaf culture, while the latter subscribes to a culturally hearing identity. As a result, those who assume Deaf identities do not view their HL as a medical issue or disability, use sign language as a first language, uphold values of Deaf culture and assert themselves as a unique and distinct community in society (Israelite et al. 2002:135; McIlroy & Storbeck 2011). On the contrary, deaf persons perceive their HL a medical condition, often use assistive hearing technology, primarily use spoken language and affiliate more with hearing people and culture (McIlroy & Storbeck 2011). Although rigid, this approach to identity construction is prevalent and primarily stems from looking at HL through the lens of medical and social models of disability (Goering 2015:134–135; Kunnen 2014:497). Some researchers have attempted to broaden the scope of identity construction for persons with HL.

#### Glickman and Holcomb’s stages of deaf identity development

Researchers in psychology that sought to illustrate diversity came up with a model consisting of four developmental stages for deaf identities on the basis of people’s relation with Deaf culture (Glickman & Carey 1993). These are culturally hearing, which foster identity based on the cultures and beliefs of the hearing community and culturally marginal identities, which exhibit confusion regarding their standpoint when it comes to the hearing and Deaf cultures (Glickman & Carey 1993). Immersion identities advocate for and have strong and uncompromising feelings about Deaf identity, and lastly, bicultural identities hold a balanced perception while still showing their deaf pride (Glickman & Carey 1993). In addition to these, Holcomb (1997:90–91) who is also deaf introduced culturally isolated and culturally separate identity categories in his model. In the former category, individuals dismiss all interaction with hearing persons, while in the latter, individuals just minimise interaction with the hearing community (Holcomb 1997:90–91).

More recently, a Bicultural DeaF identity was coined by a Bicultural DeaF researcher in disability studies for embracing identity formation in both hearing and Deaf spaces (McIlroy 2010). A participant with a bicultural DeaF identity stated that although she was deaf and felt part of the hearing culture, she had gradually opened up to a Deaf identity and was willing to forge an identity within both cultures (McIlroy & Storbeck 2011:504). In essence, the bicultural DeaF identity allows a freedom and fluidity where a strong affiliation and identification in Deaf culture can co-exist with an appreciation of identity formation in the hearing communities that one occupies. Variable deaf identities are constructed by AYA with DHL.

### The deaf identities that adolescents and young adults with disabling hearing loss construct

The experience of identity construction among AYA with DHL is a unique one. Research has shown that in comparison to their hearing peers, AYA with DHL typically embark on their deaf identity development journey earlier (Kunnen 2014:505). The formation of identity in the context of DHL is a challenging and complex process (English 2012; Kemmery & Compton 2014). Indeed, for AYA with DHL identity is layered and complex, depicting an intersection of various personal and societal factors with hearing status (Israelite et al. 2002:134). Consequently, the deaf identities that AYA with DHL construct are distinct and diverse.

#### The construction of Deaf identities

The construction of Deaf identities seems to be characterised by an early onset and sense of pride in Deaf culture. In a 5-year longitudinal study of the identity development of seven Deaf adolescents in a Netherlands school for the Deaf, researchers found that as early as 14 years, the students had the strongest commitment in the identity domain of being Deaf compared to the other identity domains such as life philosophy, friends, parents, studies and self (Kunnen 2014:505). This may have been largely influenced by the reinforcement of their cultural identity by virtue of being in a school for the Deaf. In an ethnographic study with South African young adults and adults, a participant who constructed a Deaf identity expressed pride in her cultural identity and described herself as a fully capable person (McIlroy & Storbeck 2011:504). In the same study, one participant expressed that previously, he had only perceived himself as a black Xhosa man; however, now understood being Deaf as who he is (McIlroy & Storbeck 2011:505). It was as though he now saw himself as a full embodiment of his Deaf identity. It can be deduced that the construction of Deaf identities is the direct result of a strong affiliation and immersion in Deaf culture.

#### The construction of hard-of-hearing identities

Varying experiences have been found among AYA who identify as HOH. In a phenomenological study, a participant stated that he identified as HOH and perceived himself as having a dual identity, hearing and deaf (Kemmery & Compton 2014:161). The understanding was underpinned by his ability to use both oral and signed language and experience both worlds. Similarly, in a different study with 9–16-year-old Swedish HOH adolescents, two of them constructed a bicultural HOH identity (Brunnberg 2010:8). One student explained that he regarded himself as a middleman because of his ability to cross over between hearing and deaf worlds and even preferred to socialise with hearing and deaf friends (Brunnberg 2010:11).

Moreover, a young adult in an ethnographic South African study identified as HOH, stating that he felt he was part of both hearing and deaf worlds; however, did not completely belong to either (McIlroy & Storbeck 2011). Canadian HOH adolescents solidified being HOH as a stand-alone identity that was open to interacting with the dominant hearing culture (Israelite et al. 2002:140). It seemed that the HOH identity was not only constructed from the ability to be bilingual or bicultural but, was considered as an independent identity that mediates and stands at the margins of hearing and Deaf cultures, creating a defined and unique identity.

#### The construction of hearing identities

Research has shown that some AYA will construct a hearing identity despite their HL. One adolescent in Jerusalem with a cochlear implant felt she could hear well and integrated into the hearing community (Rich et al. 2013). In a Swedish study exploring the identity of HOH adolescents, one student expressed that she related best with other hearing children, thus consciously chose not to have Deaf friends (Brunnberg 2010:8). A qualitative study that enrolled seven South African young adults with HL at a university found that they all self-identified as hearing because of an upbringing in a predominately hearing culture (Bell, Carl & Swart 2016:6). One student expressed that she had always been treated as a ‘normal’ person because her HL could not be immediately seen (Bell et al. 2016:6). Another student also expressed that they were not treated as a deaf person and went to school normally, while the other explained that he was never made to feel different in any way (Bell et al. 2016:7). It is apparent that a hearing identity among AYA with DHL is propelled by a feeling of ‘normality’ despite their DHL. This sense is brought on by being embraced and accepted as they are within the hearing community, a sense of belonging. Furthermore, the nature of HL as an ‘invisible’ disability allows AYA to integrate seamlessly within the hearing community as there is no apparent difference to distinguish them from hearing community members. Nonetheless, this integration is considerably difficult for some AYA.

#### The unresolved identities

On the other side, some AYA show an identity crisis. Adolescents and young adults who experience an identity crisis are often isolated and expressed denial and grief regarding their HL (Brunnberg 2010:9). In his introspection, one HOH adolescent constantly expressed how it would be like to be a hearing person, while one indicated that he did not want to be deaf but wanted to become hearing (Brunnberg 2010). Another adolescent not only dismissed an HOH identity but also isolated themselves from any interaction with Deaf, HOH or hearing friends, describing their situation as ‘just being a lot of trouble’ (Brunnberg 2010). Evidently, failure of AYA to reconcile the reality of their lived experiences with their wishes, coupled with little or no exploration and commitment to any identity domain can trigger an identity crisis. Negative societal attitudes, which propel stigmatisation and imposed limitations are also possible underlying reasons for this unresolved deaf identity (Suheir 2014:229). Therefore, strong feelings about wanting to be hearing may be a result of internalised stigma or reflect a faction of AYA with DHL that struggle to progress past the denial stage of grief.

#### Identities that detach from hearing loss as a disability

Furthermore, some AYA construct identities that disregard HL-related disability as a central factor in their identity construction. One study exploring the identity issues of 52 HOH adolescents in New Zealand found that most of them did not perceive themselves as having any hearing disability despite being medically and audiologically diagnosed with some level of DHL (Kent 2003:231). In relation to her HL, one participant explained that although she had a hearing problem, she did not perceive herself as disabled and would not adopt that identity (Kent 2003:231). In the same manner, seven young adults at a South African university who identified as hearing, completely discarded HL as a defining factor in their identity formation (Bell et al. 2016:6). The downside, however, was that non-disclosure of hearing status and failure to seek help for required accommodations posed a risk for their academic success (Bell et al. 2016:8).

This is exemplary of how self-constructed identities can vary greatly with externally ascribed identities and all their connotations. For instance, the diagnosis of DHL is a form of labelling that attaches the externally ascribed identity of disability, one that carries stigma, with society often perceiving disability as a liability (Murugami 2009). Given this negative societal perception, one may choose to separate their deaf identity from disability. At a personal level, perhaps disability comes secondary for these individuals and is simply not considered as a defining factor in their identity construction.

#### The construction of fluid identities

The fluidity of deaf identity is also expressed in the experiences of AYA with DHL. In a phenomenological study, HOH adolescents in the United States expressed that their identity varied depending on context (Kemmery & Compton 2014:170). In fact, one participant explained that his identity can exist on the extremes of hearing person and person with HL, where the latter identity is applicable only when their hearing aid is malfunctioning or when they attend audiological interventions (Kemmery & Compton 2014:170). This is an interesting perspective that illustrates how personalised and dynamic deaf identity construction is. It further shows how the concept of deaf identity is neither this nor that, but a spectrum with extremities and in-betweens.

The literature shows the variability and diversity of deaf identity. Could some identities be more vulnerable than others? Complex trade-offs seem to exist for some identities that AYA with DHL construct. As seen in the current literature, despite having a DHL, constructing hearing identities and identities that detach from disability may cause AYA to forgo reasonable accommodations because of non-disclosure or non-uptake of interventions to aid them, putting them in a vulnerable position (Bell et al. 2016:8). Perhaps these compromises are necessary for meeting the norms and acting within the bounds of their chosen identities helping them maintain their sense of belonging. Nonetheless, when AYA are adamant about these chosen identities, these existing trade-offs can potentially do more harm than good for personal, academic and vocational success. Similarly, AYA with unresolved identities seem vulnerable. They can be thought to be experiencing some level of identity crisis denoted by denial, low self-esteem and isolation, making them especially prone to psychopathology (Warner-Czyz et al. 2015:1). As such, AYA showing signs of identity crisis may require extensive personal adjustment counselling compared to others.

A synthesis of the literature on identity construction reveals that identity formation is not dichotomous or homogenous. Identity formation is intricate. Contrary to traditional deaf identity beliefs, AYA construct a spectrum of identities, which are driven by varying rationales. Even so, the depiction of deaf identity mainly fixates on hearing status or the cultural dynamics of hearing and Deaf communities, reinforcing a rigid view of deaf identity. Notably, exploring identity construction within the bounds of hearing status or Deaf and hearing cultural dynamics is restrictive and does not address the wider scope of identity and the psychosocial needs of AYA with DHL. Evidently, deaf identity is constructed through self-perception, engaging with disability, building relationships, adopting social roles and assimilating into communities (Bell et al. 2016; Hecht et al. 2005; Kent 2003; Kunnen 2014; McIlroy & Storbeck 2011; Pang & Hutchinson 2018). Perhaps some of the aforementioned psychosocial processes may be prioritised more than others. Although these underlying processes through which identity is constructed can be inferred from the studies, it is at a superficial level. An in-depth account of how and why AYA construct these varying identities is lacking, even more so in the African context.

## Conclusion

Contemporary discourse on deaf identity is changing, emphasising a need for portraying deaf identity in a manner that is broader and all-encompassing (Morgan & Kaneko 2017:234). In a study that explored belonging through South African Sign Language (SASL) poetry, it was stated that other than being Deaf, the identity of persons with DHL is also denoted by race, gender, sexuality, ethnicity and social class, and sometimes these identities supersede the identity of being Deaf (Morgan & Kaneko 2017:333). However, many empirical studies exploring deaf identity construction among AYA with DHL are not entirely reflective of this. Elaborate exploration of the psychosocial factors underlying the identities that AYA with DHL construct is lacking. The current studies do not explicitly highlight how AYA forge their identities, what processes underpin their self-ascribed identity choices, the trade-offs related to identity choices as well as possible challenges. When these nuances are not explored more extensively, it may undermine the vastness of identity formation and the critical processes underlying it.

The topic of identity construction is still widely under-researched from a healthcare stance and more specifically in relation to audiological practice. Identity issues are said to affect audiological outcomes and health behaviours and should be paid attention to in the care of AYA (Clark et al. 2020:55; English 2012:4). Communication function also has a direct impact on identity construction. It is imperative for rehabilitation professionals such as audiologists to be cognisant of the nuances of identity formation in the context of DHL, to provide holistic care that adequately supports the task of identity construction.

Therefore, in-depth qualitative research exploring identity construction among deaf AYA is necessary. The processes underlying deaf identity formation that encompass personal, enacted and relational identities need to be highlighted. This will make for a conceptualisation of deaf identity that is inclusive and conscious of other significant identity domains of the person. Certainly, knowing AYA’s self-ascribed identities alone is insufficient. It is also necessary to understand the inherent compromises and challenges that these identities pose and how best to mitigate them through adequate support. Further research pertaining to identity construction is pertinent in the fields exploring disability and rehabilitation where there is a constant striving to provide person-centred and holistic care that acknowledges and supports individuals in the diverse functions that they assume.
